# Molecular Structure of Phosphoserine Aminotransferase from *Saccharomyces cerevisiae*

**DOI:** 10.3390/ijms24065139

**Published:** 2023-03-07

**Authors:** Jiyeon Jang, Jeong Ho Chang

**Affiliations:** 1 Department of Biology Education, Kyungpook National University, 80 Daehak-ro, Buk-gu, Daegu 41566, Republic of Korea; 2 Department of Biomedical Convergence Science and Technology, Kyungpook National University, 80 Daehak-ro, Buk-gu, Daegu 41566, Republic of Korea

**Keywords:** phosphoserine aminotransferase, protein crystallography, *Saccharomyces cerevisiae*, phylogenetic relationship, crystal structure, pyridoxal 5′-phosphate, molecular protein structure, yeast, fungi

## Abstract

Phosphoserine aminotransferase (PSAT) is a pyridoxal 5′-phosphate-dependent enzyme involved in the second step of the phosphorylated pathway of serine biosynthesis. PSAT catalyzes the transamination of 3-phosphohydroxypyruvate to 3-phosphoserine using L-glutamate as the amino donor. Although structural studies of PSAT have been performed from archaea and humans, no structural information is available from fungi. Therefore, to elucidate the structural features of fungal PSAT, we determined the crystal structure of *Saccharomyces cerevisiae* PSAT (*Sc*PSAT) at a resolution of 2.8 Å. The results demonstrated that the *Sc*PSAT protein was dimeric in its crystal structure. Moreover, the gate-keeping loop of *Sc*PSAT exhibited a conformation similar to that of other species. Several distinct structural features in the halide-binding and active sites of *Sc*PSAT were compared with its homologs. Overall, this study contributes to our current understanding of PSAT by identifying the structural features of fungal PSAT for the first time.

## 1. Introduction

L-serine is an essential component of various metabolic pathways, from bacteria to eukaryotes [1,2]. This amino acid is derived from the diet, protein and phospholipid degradation, and its direct synthesis from glycine. However, these means are generally insufficient to compensate for deficiencies in the serine biosynthesis pathway, which is the main source of serine [3,4]. L-serine is a precursor of methionine and cysteine in amino acid biosynthetic pathways [5]. Moreover, it plays a major role in one-carbon metabolism, which is necessary to produce 5,10-methylenetetrahydrofolate (5,10-mTHF) via L-serine hydroxymethyltransferase [6]. Sphingolipids are cell membrane constituents produced by the serine palmitoyltransferase-catalyzed reaction between palmitoyl-CoA and L-serine, and phosphatidylserine is formed by phosphatidylserine synthase [7,8].

L-serine biosynthesis proceeds mainly via the phosphorylated pathway, which is conserved in plants and mammals [9]. The phosphorylated pathway of serine biosynthesis (PPSB) commences with the NAD^+^-dependent oxidation of 3-phosphoglycerate (3-PG) to 3-phosphohydroxypyruvate (3-PHP) by 3-PG dehydrogenase (PGDH) [10]. In the following step, phosphoserine aminotransferase (PSAT) catalyzes the transfer of amino groups from glutamate to 3-PHP to yield 3-phosphoserine (3-PS) and α-ketoglutaric acid (α-KG) [11]. In plants, in addition to the PPSB, serine synthesis also occurs via photorespiration and the glycerate pathway [10]. In the photorespiration reaction, two glycines are converted directly to serine via two divided pathways that are involved via the glycine decarboxylase complex (GDC) and Ser hydroxymethyltransferase (SHMT). The glycerate pathway facilitates serine biosynthesis from the dephosphorylation of 3-phosphoglyceric acid (3-PGA) mediated by 3-PGA phosphatase (PGAP), glycerate dehydrogenase (GDH), and alanine-hydroxypyruvate aminotransferase and glycine hydroxypyruvate aminotransferase (AH-AT and GH-AT). Similarly, in other organisms, such as yeast and bacteria, these glycolytic reactions are catalyzed by a series of enzymes: PGDH (EC 1.1.1.95), PSAT (EC 2.6.1.52), and 3-phosphoserine phosphatase (PSP, EC 3.1.3.3) [12,13,14,15,16]. Mice with a PGDH gene deletion and human patients with a mutation in the PPSB gene show reduced serine levels in the brain and blood, respectively, indicating the involvement of PPSB in mammalian serine biosynthesis [17,18]. This genetic evidence suggests that the phosphorylated pathway is the only route for serine biosynthesis from glucose in all non-photosynthetic organisms [11].

The pyridoxal-5′-phosphate (PLP)-dependent transamination reaction catalyzed by PSAT, which necessitates a lysine residue in the active site, has two reversible half-reactions [19,20]. In *Arabidopsis thaliana* PSAT (*At*PSAT), the internal aldimine is initially protonated at Nζ of the lysine residue (K265 in *At*PSAT) by the hydroxyl group of PLP. Following this reaction, the amine group of L-Glu carries out a nucleophilic attack on the iminium carbon of the internal aldimine. Subsequent to the PLP-Glu geminal state, a PLP-external aldimine is created, and lysine Nζ exits the active site [5].

In humans, PSAT is encoded by the *PSAT1* gene (OMIM 610936), which has two splice variants, namely PSATα and PSATβ, which were previously cloned from a human Jurkat T-cell cDNA library [21]. The PSATα transcript lacks exon 8 and encodes a protein containing 324 amino acids, whereas PSATβ includes a full-length transcript and encodes 370 amino acids. In addition, PSATβ shares 93.5% amino acid similarity with rabbit homologs and 36.5% similarity with *Saccharomyces cerevisiae* PSAT [22]. In yeast *S. cerevisiae*, the SER1 gene encodes PSAT and yeast cells supported by fermentable carbon sources, mainly by the glycolytic pathway, to synthesize serine and glycine [23]. The yeast SER1’s transcription is regulated by amino acid biosynthesis mediated by GCN4, which binds to the SER1 promoter and GCN4 recognition elements (GCREs). Industrially, the overexpression of SER genes in *S. cerevisiae* improves pharmaceutically used glutathione production via metabolic engineering of the L-serine biosynthetic pathway [24,25,26].

The structure of PSAT was first elucidated from *Escherichia coli* [1]. Since then, several PSAT structures have been identified in bacteria and protists [1,5,20,27,28,29]. Moreover, the crystal structure of PSAT has recently been elucidated from *A. thaliana* [5]. Nevertheless, the structural features of PSAT in yeast have not yet been reported. Therefore, we aimed to determine the crystal structure of PSAT from *S. cerevisiae* and identify species-specific conformational differences.

## 2. Results

### 2.1. Overall Structure of ScPSAT

**Figure 1:**
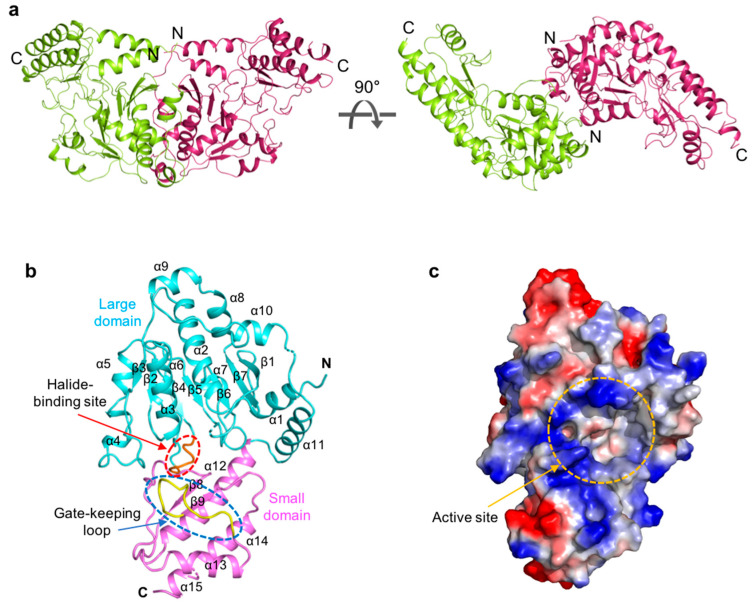
Overall structure of *Saccharomyces cerevisiae* phosphoserine aminotransferase (*Sc*PSAT). (**a**) Schematic drawing of the dimeric structure of *Sc*PSAT. The two monomers are indicated in green and purple, respectively. The right panel depicts 90° rotation along the X-axis. (**b**) The monomeric structure of *Sc*PSAT consisting of the large domain (cyan), small domain (pink), gate-keeping loop (yellow, highlighted with a blue-dashed oval), and halide-binding site (red, highlighted with a red-dashed oval). (**c**) Electrostatic surface representation of the *Sc*PSAT monomer, indicating the negatively charged (red) and positively charged (blue) surfaces and the active site (orange-dashed circle).

*Sc*PSAT exists as a dimer in the asymmetric unit and appears butterfly-shaped (Figure 1a). Fifty-three N-terminal and seven internal residues (i.e., Lys 218, Asn 219, Ile 220, Ser 268, Ala 269, Tyr 270, and Asn 271) could not be modeled due to weak electron density. The *Sc*PSAT monomer consisted of two domains: a large domain (residues 54–293) and a small domain (residues 294–395) (Figure 1b). Furthermore, the large domain exhibited a Rossmann fold that contained central seven-stranded β-sheets (β1–7 with strand topology 3-2-4-5-6-7-1) surrounded by twelve α-helices at the periphery of the domain. All strands, except for strand β7, were parallel. The small domain folded into antiparallel β-strands, β8 and β9, flanked by four α-helices, α12, α13, α14, and α15 (Figure 1b). Moreover, the halide-binding site and gate-keeping loop were located between the large and small domains. The large cavity between the two domains included an active site pocket surrounded by a positively charged cluster (Figure 1c). This region indicated the binding site of the negatively charged cofactor PLP.

### 2.2. Structural Comparisons between ScPSAT and Its Homologs

Structural homologs of *Sc*PSAT were identified using the Dali server [30]. The output coordinates revealed that the homologous structures of *Sc*PSAT were mainly from bacteria, plants, and mammals (Table 1). The structure of *Sc*PSAT was highly similar to that of *Homo sapiens* PSAT (*Hs*PSAT; PDB code: 3E77) and *At*PSAT (PDB code: 6CZX). *Hs*PSAT and *At*PSAT had a root mean square deviation (RMSD) of 1.7 Å and Z-scores of 35.4 and 35.5, respectively. Moreover, the bacterial PSAT homologs were also highly comparable to *Sc*PSAT. The PSAT structures of the well-known pathogenic bacterium *Pseudomonas aeruginosa* (*Pa*PSAT; PDB code: 4XK1) and Gram-negative bacterium *Alkalihalobacillus alcalophilus* (*Aa*PSAT; PDB code: 1W23) had RMSDs of 1.6 Å and 1.9 Å, and Z-scores of 35.5 and 34.5, respectively. However, *Sc*PSAT has relatively low homology to the PSAT of *E. coli* (*Ec*PSAT; PDB code: 1BJO), which had an RMSD of 2.3 Å and a Z-score of 34.1.

**Figure 2:**
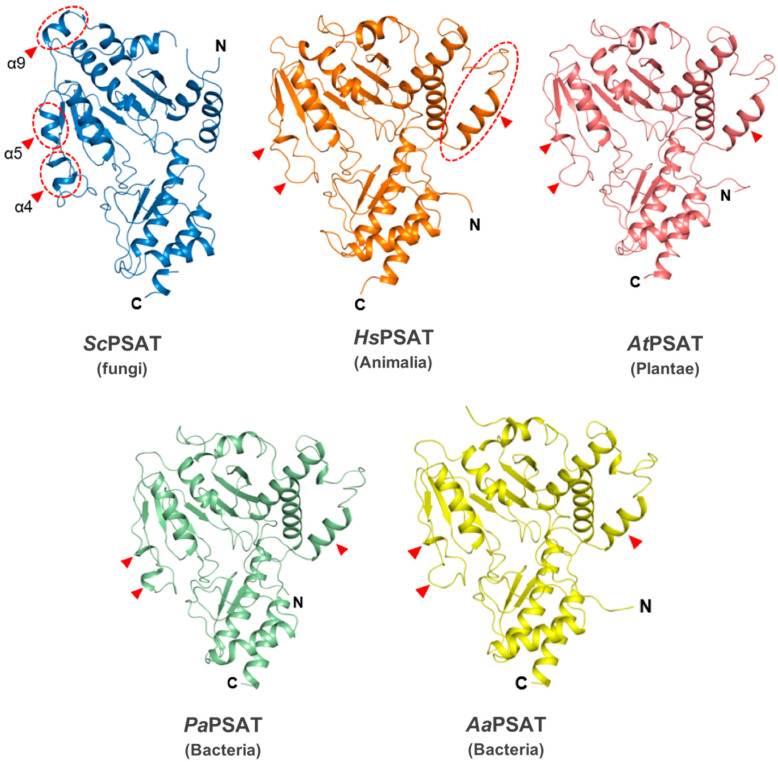
Structural comparisons of *Sc*PSAT with its homologs. Overall structures of *Sc*PSAT (blue), *H. sapiens* PSAT (*Hs*PSAT; PDB code: 3E77; orange), *A. thaliana* PSAT (*At*PSAT; PDB code: 6CZX; pink), *P. aeruginosa* PSAT (*Pa*PSAT; PDB code: 4XK1; green), and *A. alcalophilus* PSAT (*Aa*PSAT; PDB code: 1W23; yellow). Secondary structural elements α4, α5, and α9 helices of *Sc*PSAT are marked accordingly. Red-dashed ovals and red arrowheads indicate the positions of distinct conformations.

**Figure 3:**
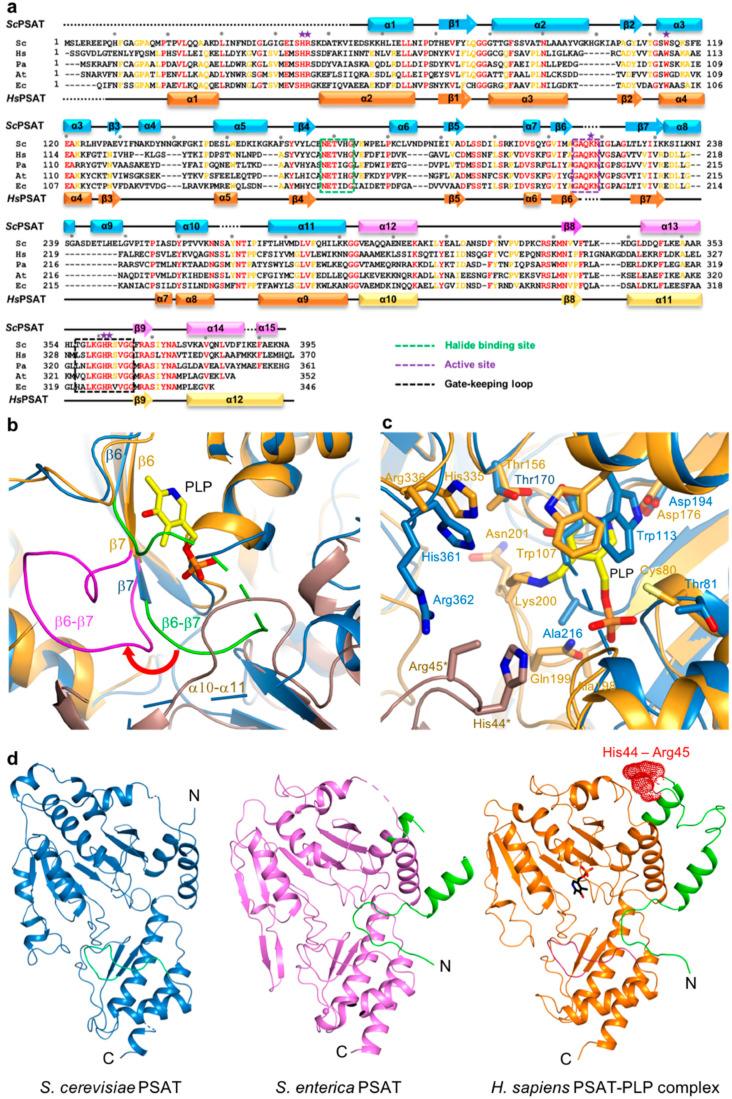
Active site of *Sc*PSAT. (**a**) Sequence alignment of *Sc*PSAT (KAF4004669.1), *Hs*PSAT (NP_478059.1), *Pa*PSAT (WP_003106701.1), *At*PSAT (NP_195288.1), and *E. coli* PSAT (*Ec*PSAT; WP_000057138.1). Secondary small domain (pink) and large domain (cyan) structural elements of *Sc*PSAT are presented on the top. Conserved residues with 100% identity among the indicated species are highlighted in red. Conserved residues with 80% identity are colored in orange. Purple stars above the sequences indicate residues involved in the catalytic reaction. Black dots above the sequences indicate every tenth residue. Active site, gate-keeping loop, and halide-binding site are marked by black-dotted squares. (**b**) Schematic drawing of the overlaid active site of *Sc*PSAT (blue) and *Hs*PSAT (orange). A neighboring molecule of *Hs*PSAT is shown in brown color. Pyridoxal-5-phosphate (PLP) is colored yellow. The β6–β7 loops of *Sc*PSAT and *Hs*PSAT are highlighted by green and magenta colors, respectively. Conformational change of β6–β7 is indicated by the red arrow. (**c**) Detailed view of the active site by superimposed structures of *Sc*PSAT (blue) and *Hs*PSAT (orange). Residues His44 and Arg45 are from a neighboring molecule of *Hs*PSAT. Lys218, His45, and Arg46 of *Sc*PSAT (equivalent to His44 and Arg45 of *Hs*PSAT) could not be modeled due to weak electron density. PLP is colored yellow. (**d**) Overall structures of PSAT from *Saccharomyces cerevisiae*, *Salmonella enterica* (PDB code: 3QM2), and *Homo sapiens* (PDB code: 3E77). The N-terminal 53 residues are colored green. His44 and His45 in the HsPSAT-PLP complex are indicated by red-dotted surface representation.

The overall structure of *Sc*PSAT relative to each of its homologs was similar at the Rossmann folds and C-terminal small domains. However, the detailed conformations of the substrate-binding domain showed several distinct features (Figure 2). For instance, helix α9 existed only in *Sc*PSAT, whereas human, plant, and bacterial structures exhibited an unstructured loop. In addition, the *Sc*PSAT structure contained α4 and α5 helices, which were replaced by incomplete helices or small loops in the homologous PSAT structures. The additional helices in the *Sc*PSAT structure could be attributed by insertional sequences (Figure 3a). Notably, the homologous PSAT structures contained an additional helix at the N-terminus, which was absent in that of *Sc*PSAT. Furthermore, the positions of α-helices and β-sheets in the C-terminal small domain exhibited slight species-specific differences.

### 2.3. Active Site

The active site of PSAT was optimized for the binding of L-Glu, 3-PHP, α-KG, and 3-PS. According to *Hs*PSAT and *At*PSAT, six conserved residues (i.e., His45*, Arg46*, His361, Arg362, Trp113, and Lys218; asterisks indicate residues from the other subunit within the dimer) were involved in catalytic activity (Figure 3a). The histidine and arginine residues were related to 3-PS binding, and His45 and Arg46 formed phosphate-binding sites in the active site [5]. Moreover, the cofactor PLP is π-stacked with a Trp residue, which is necessary for the complete enzymatic reaction [31].

The structures of the *Sc*PSAT and *Hs*PSAT-PLP complex were overlaid to compare their active sites. Notably, the β6–β7 loop of *Hs*PSAT exhibited a large conformational change approximately 9 Å outward from PLP, which may be attributed to PLP binding (Figure 3b). The α10–α11 loop of *Sc*PSAT showed partially weak electron density due to its flexibility, whereas that of *Hs*PSAT was clearly shown near the phosphate group of PLP. This may be because PLP binding stabilizes the loop.

A detailed view of the active site revealed that the overlaid residues were mainly located at similar positions (Figure 3c). However, PLP binding on *Hs*PSAT resulted in distinct side chain conformations compared with those of *Sc*PSAT. Moreover, Trp107 of *Hs*PSAT corresponded to Trp113 of *Sc*PSAT and rotated to form a π-stacking interaction with the pyridine ring of PLP. Lys200 of *Hs*PSAT formed a Schiff base aldimine linkage with PLP, whereas Lys218 of *Sc*PSAT was disordered. Furthermore, the active site also comprised His44 and Arg45 of the neighboring *Hs*PSAT subunit; however, they were not visible in *Sc*PSAT. We confirmed that no partial degradation of the *Sc*PSAT protein occurred during crystallization (Figure S1). These distinct conformations of the active site residues are also shown relative to the other homologous structures (Figure S2).

**Figure 4:**
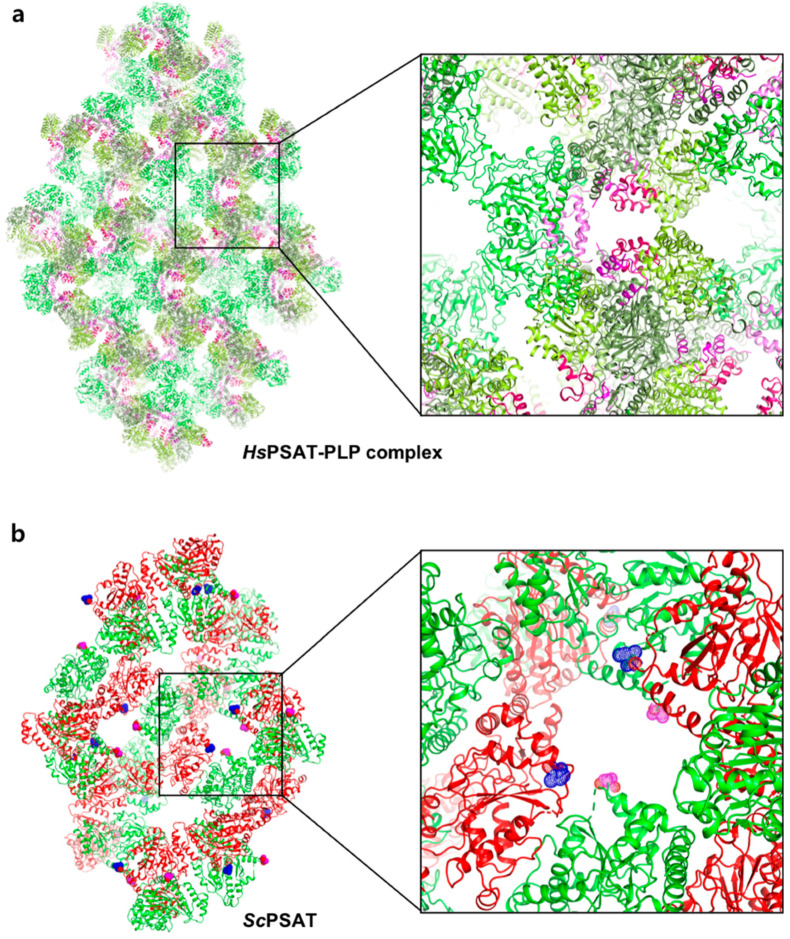
Comparison of the crystallographic symmetry of *Hs*PSAT-PLP complex and *Sc*PSAT. (**a**) Each of the trimeric *Hs*PSAT-PLP complex subunits is colored in deep green, green, and light green. The N-terminal fragments are colored pink, magenta, and red. One of the amplified interfaces from the symmetry is shown in the right panel. (**b**) Each of the dimeric *Sc*PSAT subunits is colored in green and red. The N-termini from the subunits are highlighted by magenta and blue-dotted spheres.

**Figure 5:**
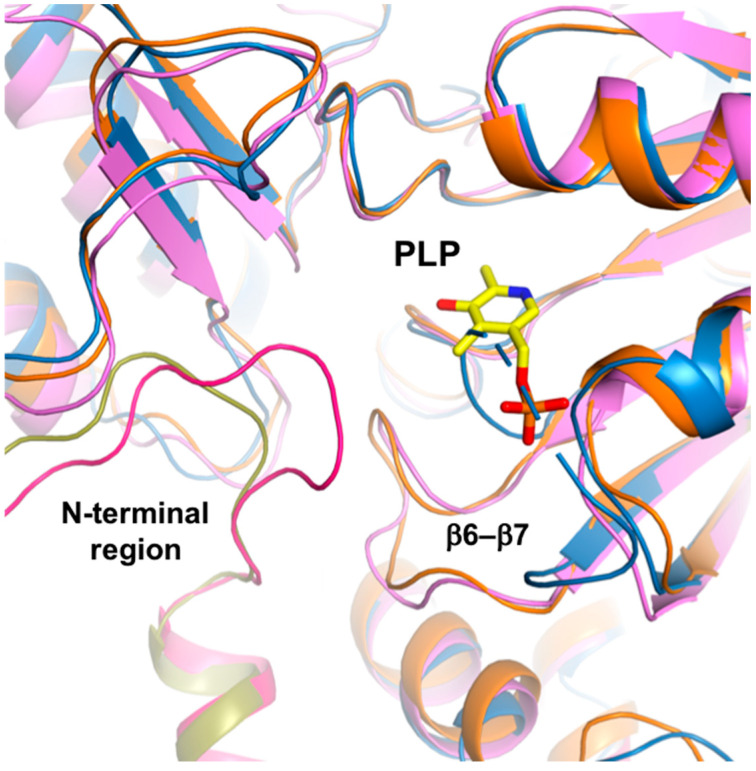
N-terminal comparison. Overlaid structures of *Sc*PSAT (blue), *Se*PSAT (pink), and *Hs*PSAT-PLP complex (orange). While the N-terminal conformation of ScPSAT is disordered, those of *Se*PSAT and the *Hs*PSAT-PLP complex are shown in magenta and olive colors, respectively. PLP-bound in *Hs*PSAT is colored yellow.

The *Hs*PSAT structure contains a cofactor PLP, whereas the *Sc*PSAT structure is an apo-form. To assess whether the PLP binding affects the stabilization of the N-terminal region including His44 and Arg45, we compared the *Sc*PSAT with an apo-form of PSAT from *Salmonella enterica* (SePSAT) (Figure 3d). The corresponding residues of His44 and Arg45 in *Hs*PSAT of *Se*PSAT were also disordered due to flexibility. This probably indicates that the conserved His44 and Arg45 could be involved in stabilizing PLP. We also compared the crystallographic packings of both *Hs*PSAT and *Sc*PSAT (Figure 4). The N-terminal region of *Hs*PSAT showed no direct interaction with a symmetry-related molecule. This indicated that the N-terminal part was not stabilized by the packing effect but by PLP binding. Moreover, His44 and Arg45 were involved in constructing the active site of the neighboring molecule of the dimer. Due to the lack of PLP bound in *Sc*PSAT, the N-terminal region might be not stabilized in the structure. In addition, Lys218 in the β6–β7 loop was also disordered in the apo structure of *Sc*PSAT (Figure 3b). Although the conformation of the β6–β7 loop is not changed in *Se*PSAT, a part of the N-terminal region exhibited a distinct conformation compared to the *Hs*PSAT-PLP complex (Figure 5). This feature may indicate the correlation of the β6–β7 loop (Lys218) and N-terminal region (His44 and Arg45). Overall, based on the comparison of the *Sc*PSAT, *Se*PSAT, and *Hs*PSAT-PLP complex, PLP binding promotes the conformation of the active site to efficiently recognize the substrate.

### 2.4. Gate-Keeping Loop

**Figure 6:**
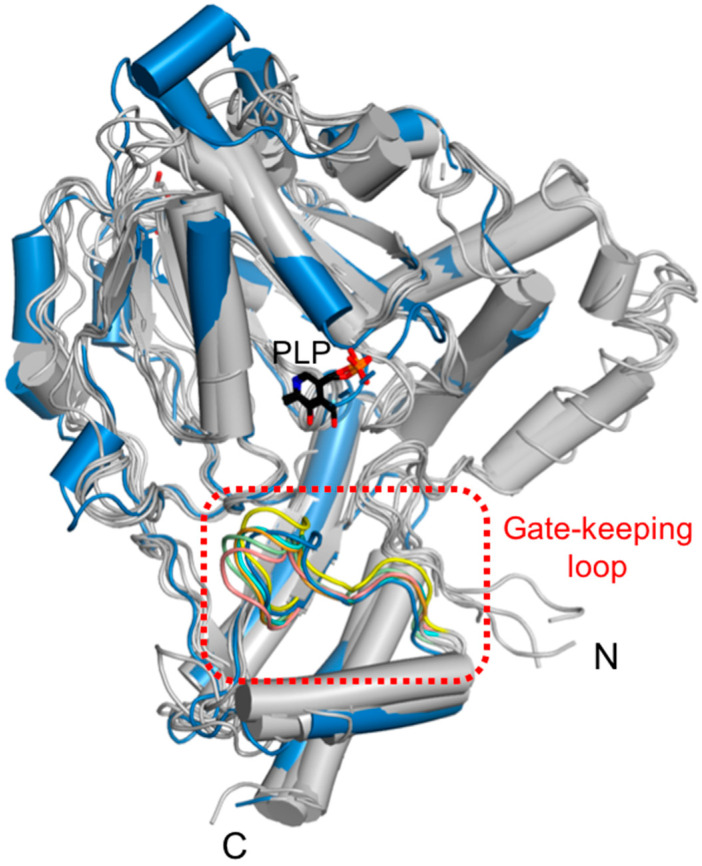
Structural comparison of the gate-keeping loop. Overlaid structures of *Sc*PSAT (blue) with its five homologous structures (grey) indicating the gate-keeping loops (red-dashed square). The gate-keeping loops of *Sc*PSAT (blue), *Hs*PSAT (PDB code: 3E77; orange), *At*PSAT (PDB code: 6CZX; pink), *Entamoeba histolytica* PSAT (*Eh*PSAT; PDB code: 5YBO; green), *Aa*PSAT (PDB code: 1W23; yellow), *Stenotrophomonas maltophilia* PSAT (*Sm*PSAT; PDB code: 6XDK; cyan), and PLP bound in *Eh*PSAT (black) are visualized.

Eleven conserved residues in a part of the α13-β9 loop of the small domain were located in the vicinity of the *Sc*PSAT active site. This loop, called the gate-keeping loop, covers the active site and facilitates the accurate positioning of the bound PLP. In *Sc*PSAT, the gate-keeping loop is represented by amino acid residues spanning Thr356 to Gly366, which correspond to that of *Hs*PSAT spanning Leu330 to Gly 340 (Figure 3a). The gate-keeping loops in the structures of *Sc*PSAT, *Stenotrophomonas maltophilia* PSAT (*Sm*PSAT), *At*PSAT, *Aa*PSAT, *Entamoeba histolytica* PSAT (*Eh*PSAT), and the *Hs*PSAT-PLP complex showed similar conformations (Figure 6). In the compared structures, the conformation of the *Sc*PSAT gate-keeping loop was distinct from those of *At*PSAT and *Eh*PSAT (Figure S3).

### 2.5. Putative Halide-Binding Site

**Figure 7:**
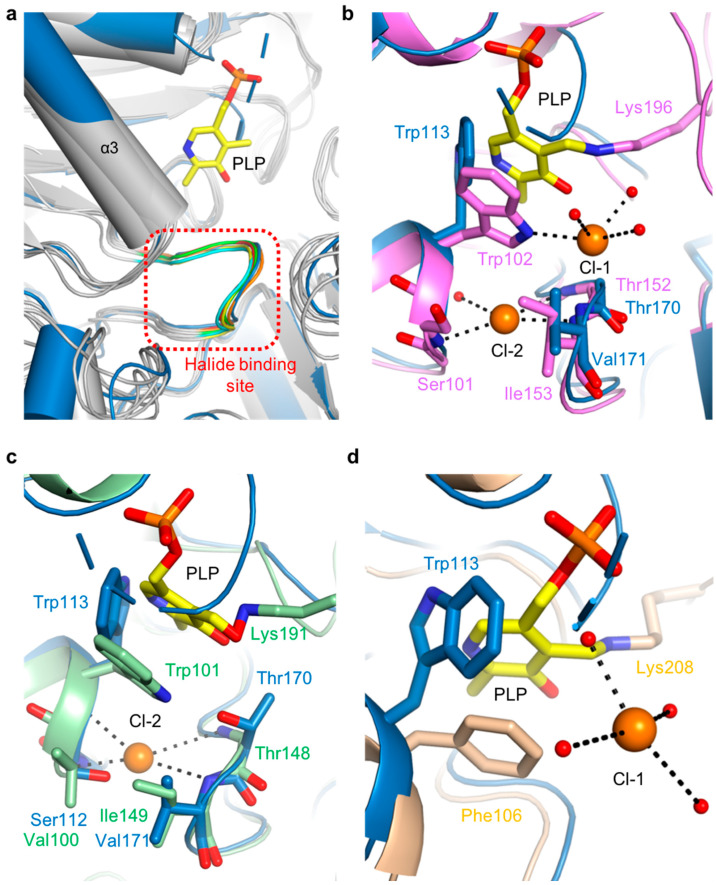
Comparison of halide-binding sites. (**a**) Overlaid structures of *Sc*PSAT (blue) with its five homologs (grey) indicating the halide-binding site (red-dashed square). The loops of *Sc*PSAT (blue), *Hs*PSAT (PDB code: 3E77; orange), *At*PSAT (PDB code: 6CZX; red), *Pa*PSAT (PDB code: 4XK1; green), *Aa*PSAT (PDB code: 1W23; yellow), and *Ec*PSAT (PDB code: 1BJO; cyan), and the PLP bound in *Aa*PSAT (yellow) are visualized. (**b**) Detailed view of halide-binding site by superimposed structures of *Sc*PSAT (blue) and *Aa*PSAT (pink). Two chloride ions (Cl-1 and Cl-2; orange), coordinated water molecules (red), and PLP bound by *Aa*PSAT (yellow) are shown. (**c**) Detailed view of halide-binding site by superimposed structures of *Sc*PSAT (blue) and *Eh*PSAT (green). Cl-2 (orange) and PLP bound by *Eh*PSAT (yellow) are shown. (**d**) Detailed view of halide-binding site by superimposed structures of *Sc*PSAT (blue) and *Pyrococcus horikoshii* PSAT (*Ph*PSAT; PDB code: 2DR1; light brown). Cl-1 (orange), coordinated water molecules (red), and PLP bound by *Ph*PSAT (yellow) are shown.

The putative halide-ion-binding site in *Sc*PSAT covered residues 168–173 in a part of the β4-α6 loop (Figure 3a). This region followed the motif NX_1_TX_2_X_3_G, with X_1_ found to be either Asn or Glu, X_2_ either Ile or Val, and X_3_ being variable. This region had a near-identical conformation to that of the other homologous structures (Figure 7a). Although no metal ion density was found in the putative halide-binding site of *Sc*PSAT, only four homologous structures, *Aa*PSAT (PDB code: 1W23), *Eh*PSAT (PDB code: 5YB0), *Pyrococcus horikoshii* PSAT (*Ph*PSAT; PDB code: 2DR1), and *Sm*PSAT (PDB code: 6XDK), have been shown with bound chloride ions. However, the chloride ions in *Sm*PSAT were located too far from the active site so that it may have a distinct mechanism, as shown in angiotensin-I converting enzyme (ACE) (Figure S4) [32]. Therefore, three structures of *Aa*PSAT, *Eh*PSAT, and *Ph*PSAT were superimposed for analysis (Figure 7b–d).

*Aa*PSAT contained two chloride ions. The first chloride ion (Cl-1) was coordinated by Trp102 and three water molecules, and the second chloride ion (Cl-2) was coordinated by the main chains of Ser101, Thr152, and Ile153 (Figure 7b). The residues neighboring Cl-2 were superimposed well, whereas Trp113 of *Sc*PSAT had a distinct conformation due to the unbound PLP. The *Eh*PSAT-PLP complex contained one chloride ion at a position corresponding to the Cl-2 position of *Aa*PSAT (Figure 7c). Similar to *Aa*PSAT, the residues Val100, Thr148, and Ile149 surrounding Cl-2 of *Eh*PSAT were well superposed with those corresponding to *Sc*PSAT. The position of the bound chloride ion in *Ph*PSAT was similar to that of Cl-1 (Figure 7d). In contrast to Cl-1 of *Aa*PSAT, that of *Ph*PSAT had no interaction with the protein and was coordinated with four water molecules. Moreover, Trp113 was replaced with Phe106 in *Ph*PSAT.

## 3. Discussion

PLP-dependent enzymes are abundant and include classes such as transferases, lyases, and isomerases [33,34,35]. These enzymes demonstrate significant functional diversity, indicated by over 140 unique enzymatic activities that account for approximately 4% of all classified enzyme activities [34]. PSAT is a PLP-dependent aminotransferase that catalyzes the transfer of an amino group from Glu to 3-PHP in the serine synthesis pathway to yield 3-PS and α-KG [10]. Although several PSAT structural studies have been conducted in humans, plants, and bacterial species, little is known about PSAT in fungal species [1,5,20,27,28]. In this study, we observed that the overall structure of *Sc*PSAT mainly adopted a fold similar to that of its homologous proteins; however, the N-terminal domain exhibited considerable conformational variation in the α1 and α9 helical regions. None of the structurally homologous proteins from bacteria (*Pa*PSAT and *Aa*PSAT), plants (*At*PSAT), and mammals (*Hs*PSAT) had a helical conformation corresponding to that of α9 in *Sc*PSAT. However, there was no helix in *Sc*PSAT corresponding to α11 and α12 in *Hs*PSAT because fifty-three residues (1–53) were not modeled in *Sc*PSAT. These conformational differences demonstrated the unique features of *Sc*PSAT compared to its homologous proteins. Furthermore, *Sc*PSAT existed as a dimer in the asymmetric unit, and the dimeric configuration of PSAT is fundamental to its functional activity [20]. Similar to the PSAT homologs, the *Sc*PSAT monomer consisted of two domains. The large N-terminal domain (residues 54–293 in *Sc*PSAT), including a halide-binding site and a PLP-binding site, adopted a typical Rossmann-fold motif with high sequence identity, whereas the small domain (residues 294–395 in *Sc*PSAT), including the gate-keeping loop, was slightly variable.

The catalytic residues in the *Sc*PSAT structure were highly conserved, and hydrogen bonds between 3-PS and four residues (His45, Arg46, His361, and Arg362) were essential for the enzymatic activity [28]. Thus, the indole ring of Trp113 is important for stacking interactions with PLP and offers additional stabilization via a hydrogen bond with PLP [20,27]. Moreover, fluorescence resonance energy transfer (FRET) studies suggested that the orientation of Trp113 changes such that it approximates to PLP owing to changes in pH [36]. In addition, the electron sink property of PLP acts on Lys218, which forms an internal aldimine between PLP and Lys known as an LLP moiety [37,38]. The strain and distortion of the conjugated π-electron system of PLP and the internal aldimine in PSAT are essential for the entire enzyme reaction [39]. Notably, the 53 N-terminal residue-truncated *Sc*PSAT may have largely diminished catalytic activity. According to a previous study, the deletion of 45 N-terminal residues results in an inactive *Eh*PSAT [20]. Moreover, the deletion of 15 and 4 N-terminal residues resulted in 98% and 90% decreased activity, respectively. In *At*PSAT, approximately 70 amino acids at the N-terminus are transit peptides that exist in chloroplasts in plant cells [40].

The gate-keeping loop, which is generally found in other related enzymes, plays a crucial role in recognizing the substrate and stabilizing the active site by inducing a closed conformation [41,42,43]. However, little is known about the gate-keeping loops of PSAT enzymes. The complete catalytic activity of aminotransferases likely necessitates a closed conformation [44]. Considering this, it may be essential that the gate-keeping loop of *Sc*PSAT induces an optimal conformation with His361. Moreover, Arg362 may be positioned closer to the PLP-binding site to direct the substrates in the proper position. The halide-binding site, which is an NX_1_TX_2_X_3_G motif, is also widely found in PSAT structures [20,28,45]. The putative halide-binding site of *Sc*PSAT was 168-NETVHG-173. In particular, Thr170 was involved in the catalytic reaction by interacting with both PLP and the chloride ions. Trp113 was located near the chloride ion, suggesting its role in stabilizing the ion, which may explain the halide-dependent inhibition of *Sc*PSAT activity [27].

Further studies are required to validate the accurate molecular mechanism of *Sc*PSAT. First, mutagenesis of Trp113 to Ala, Phe, and His could confirm the role of Trp in the activity of *Sc*PSAT, such as the indole ring stacking of Trp with the cofactor PLP. Second, it would be worthwhile to demonstrate the effects of pH and halide ions on the active sites. According to a previous study, NaCl and NaBr inhibited enzymatic activity, whereas NaF did not show any effect on *Eh*PSAT [36]. Conversely, the activity of *Tv*PSAT is not inhibited by NaF, NaCl, or NaBr, but only by NaI [27]. In other words, sodium halides do not consistently inhibit PSAT in different species. Hence, it is necessary to perform kinetic measurements of *Sc*PSAT to elucidate the inhibitory effects of sodium halides. Finally, additional trials are necessary to obtain the complex structure of *Sc*PSAT with PLP, PMP, and 3-PS to elucidate the binding mode based on conformational changes.

Taken together, the crystal structure and structure-based phylogenetic analysis of *Sc*PSAT may contribute to advancing our knowledge of the overall view of the various PSAT enzymes.

## 4. Materials and Methods

### 4.1. Preparation of PSAT Expression Constructs

Full-length PSAT genes of *S. cerevisiae* (*Sc*PSAT; NCBI ID: AJT97493.1) were acquired. The yeast PSAT-encoding genes were amplified by PCR using genomic DNA obtained from the Korean Collection for Type Cultures as a template, Pfu-X DNA polymerase (SolGent, Daejeon, Republic of Korea), and oligonucleotide primers (Cosmo Genetech Inc., Seoul, Republic of Korea). The amplified products were digested with appropriate restriction enzymes (Enzynomics, Daejeon, Republic of Korea) at 37 °C for 3 h. The digested products were ligated with the pET28a vector using T4 ligase (M0202S; Roche, Basel, Switzerland) at 18 °C overnight and subsequently transformed into *E. coli* strain DH5α. The transformants were confirmed by colony PCR and DNA sequencing.

### 4.2. Purification of Recombinant Proteins

For the overexpression of N-terminal Hisx6-tagged *Sc*PSAT, plasmids encoding *S. cerevisiae* PSAT were transformed into *E. coli* BL21 (DE3) Star. Cells were grown in Luria–Bertani medium (Ambrothia, Deajeon, Republic of Korea) containing 50 mg/L kanamycin (AppliChem, Darmstadt, Germany) at 37 °C until the optical density at 600 nm (OD_600_) reached approximately 0.6. Following induction with 0.3 mM isopropyl β-D-1-thiogalactopyranoside (IPTG; Calbiochem, Burlington, MA, USA), the cells were incubated for 16 h at 20 °C. Subsequently, cultured cells were harvested by centrifugation at 4000 rpm for 20 min at 4 °C. The cell pellet was resuspended in a buffer containing 20 mM Tris (pH 8.0) (Sigma-Aldrich, St. Louis, MO, USA), 250 mM NaCl (AppliChem, USA), 5% glycerol (Affymetrix, Santa Clara, CA, USA), 0.2% Triton X-100 (Sigma-Aldrich, USA), 10 mM β-mercaptoethanol (Bio Basic, Markham, ON, Canada), and 0.2 mM phenylmethylsulfonyl fluoride (PMSF; Sigma–Aldrich). Cells were disrupted via ultrasonication (Sonics VCX-500/750, Sonics and Materials Inc., Newtown, CT, USA) for 15 min with 3 s pulse-on and 3 s pulse-off cycles. Cell debris was removed by centrifugation at 13,000 rpm for 40 min, and the supernatant was bound to Ni–NTA agarose (Qiagen, Hilden, Germany) for 90 min at 7 °C. After washing with a buffer containing 5 mM imidazole (Sigma-Aldrich, USA), the bound proteins were eluted with 50 mM Tris (pH 8.0), 200 mM NaCl, and 250 mM imidazole. Size exclusion chromatography (SEC) was performed using HiPrep 16/60 Sephacryl S-300 HR (GE Healthcare, Chicago, IL, USA) with a buffer containing 20 mM Tris (pH 7.5), 150 mM NaCl, and 2 mM dithiothreitol (DTT; Calbiochem, USA). Following SEC, *Sc*PSAT proteins were stored at −80 °C for subsequent crystallization trials. Purified proteins were assessed by SDS-PAGE using a 15% acrylamide gel.

### 4.3. Crystallization and Improvements

All crystallization trials were performed at 20 °C using the sitting-drop vapor diffusion method in 96-well sitting-drop vapor diffusion crystallography plates (Art Robbins Instruments, Sunnyvale, CA, USA). Over 600 different conditions from sparse-matrix screening solution kits were tested to identify optimal crystallization conditions. Rod-shaped *Sc*PSAT crystals were obtained over a week from 9.2% (*v*/*v*) Tacsimate^TM^ (pH 5.0) and 16.5% (*w*/*v*) PEG 3350 using sparse-matrix screening. To improve the crystals, additional screening was performed using additive (HR2-428, Hampton Research, Viejo, CA, USA) and detergent (HR2-406, Hampton Research, USA) screening. The optimized crystals were obtained from 2.4 M ammonium sulfate and 0.1 M citric acid (pH 3.5), and the average size of the crystals was 200 × 50 × 50 μm.

### 4.4. Data Collection and Structure Determination

Prior to data collection, the crystals were flash-cooled in liquid nitrogen under the crystallization conditions, supplemented with 30% (*v*/*v*) ethylene glycol. All diffraction datasets were collected at 100 K on beamline 5C of the Pohang Accelerator Laboratory, Republic of Korea, using a Quantum 315r CCD detector (Area Detector Systems Corporation, Poway, CA, USA). Data were processed using the HKL-2000 software suite. The *Sc*PSAT crystals belonged to space group *P*3_1_21 and diffracted to a resolution of 2.8 Å. The experimental electron density maps were obtained by molecular replacement methods using PHENIX software, version 1.9, and interpreted using the WinCoot program (PHENIX, Berkeley, CA, USA). *At*PSAT (PDB code: 6CZX) served as the search model (Table 2).

## Figures and Tables

**Table 1:** Structural similarity between *Sc*PSAT and its homologs, compared using Dali ^a^.

Species	*Z*-Score	RMSD (Å)	Identity (%)	C_α_	PDB Code
*Homo sapiens*	35.4	1.7	42	362	3E77
*Pseudomonas aeruginosa*	35.5	1.6	41	355	4XK1
*Arabidopsis thaliana*	35.5	1.7	41	361	6CZX
*Alkalihalobacillus alcalophilus*	34.5	1.9	38	360	1W23
*Escherichia coli*	34.1	2.3	37	360	1BJO

^a^ This server computes optimal and suboptimal structural alignments of two protein structures using the DaliLite pairwise option. http://ekhidna.biocenter.helsinki.fi/dali/.

**Table 2:** Data collection and refinement statistics for *Sc*PSAT.

Statistics	*Sc*PSAT
Data collection	
Space group	*P*3_1_21
a, b, c (Å)	132.28, 132.28, 141.62
α, β, γ (°)	90, 90, 120
Resolution range (Å) ^a^	48.3–2.8 (2.87–2.80)
No. of total reflections	478,593
No. of unique reflections	68,146
Completeness (%)	100 (100)
*I*/σ (*I*)	33.6 (6.9)
*R*_merge_ (%) ^b^	13.4
CC_1/2_	0.999(0.948)
**Structure refinement**	
Resolution range (Å)	48.3–2.8
No. of reflections	35,712
*R*_work_ ^c^/*R*_free_ ^d^	22.8/26.8
R.M.S. deviation	
Bond lengths (Å)	0.010
Bond angles (°)	1.094
Average *B*-factor (Å^2^)	
Protein	62.12
Solvent	52.41
Ramachandran plot ^e^	
Most favored (%)	91.2
Additional allowed (%)	8.1
Disallowed (%)	0.7
PDB code	8I28

^a^ The numbers in parentheses are statistics from the highest-resolution shell. ^b^ $R_{\mathrm{merge}}=\sum \left|I_{\mathrm{obs}}-I_{\mathrm{avg}}\right|/I_{\mathrm{obs}}$, where *I*_obs_ is the observed intensity of individual reflections and *I*_avg_ is the averaged observed intensity over symmetry equivalents. ^c^ $R-\mathrm{factor}=\sum h\left|\left|F_{o}\left(h\right)\right|-\left|F_{c}\left(h\right)\right|\right|/\sum h\left|F_{o}\left(h\right)\right|$, where *F*_o_ and *F*_c_ are the observed and calculated structure factor amplitudes, respectively. ^d^ R-free was calculated with 5% of the data excluded from the refinement. ^e^ Categories as defined by MolProbity.

## Data Availability

Not applicable.

## Supplementary Materials

The following supporting information can be downloaded at: https://www.mdpi.com/article/10.3390/ijms24065139/s1.
