# Supplementary material for: Molecular Structure of Phosphoserine Aminotransferase from Saccharomyces cerevisiae
Source: Int J Mol Sci. 2023 Mar 7;24(6):5139. doi: 10.3390/ijms24065139 (PMC10049462; doi:10.3390/ijms24065139)
Supplement: Supplementary file 1 [file ijms-24-05139-s001.zip › ijms-2115435-supplementary.pdf]

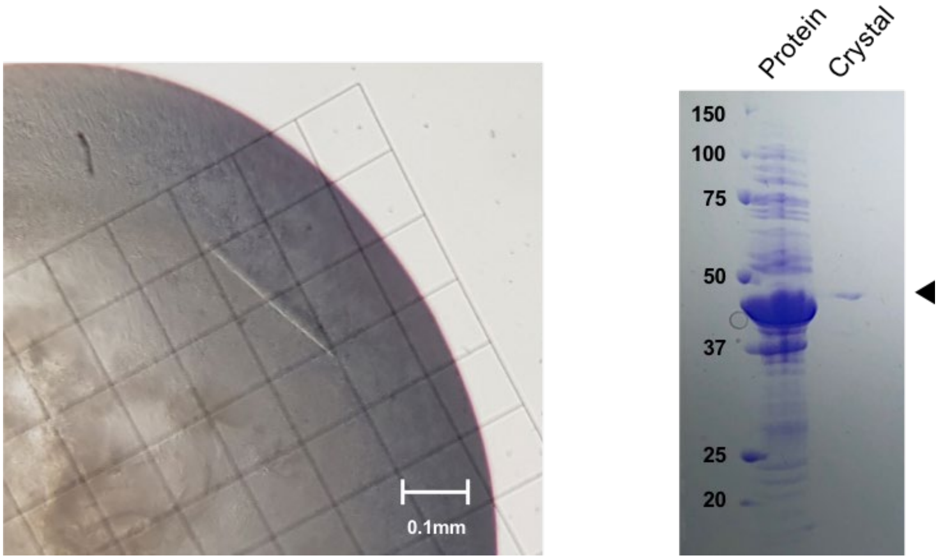

Figure S1. SDS-PAGE analysis of dissolved crystals. Crystals of *Sc*PSAT protein (left panel) were dissolved by a buffer containing 20 mM Tris (pH 7.5), 150 mM NaCl, and 2 mM dithiothreitol, and it was compared with the purified protein by using SDS-PAGE analysis (right panel). The sizes of standard marker were shown on the gel.

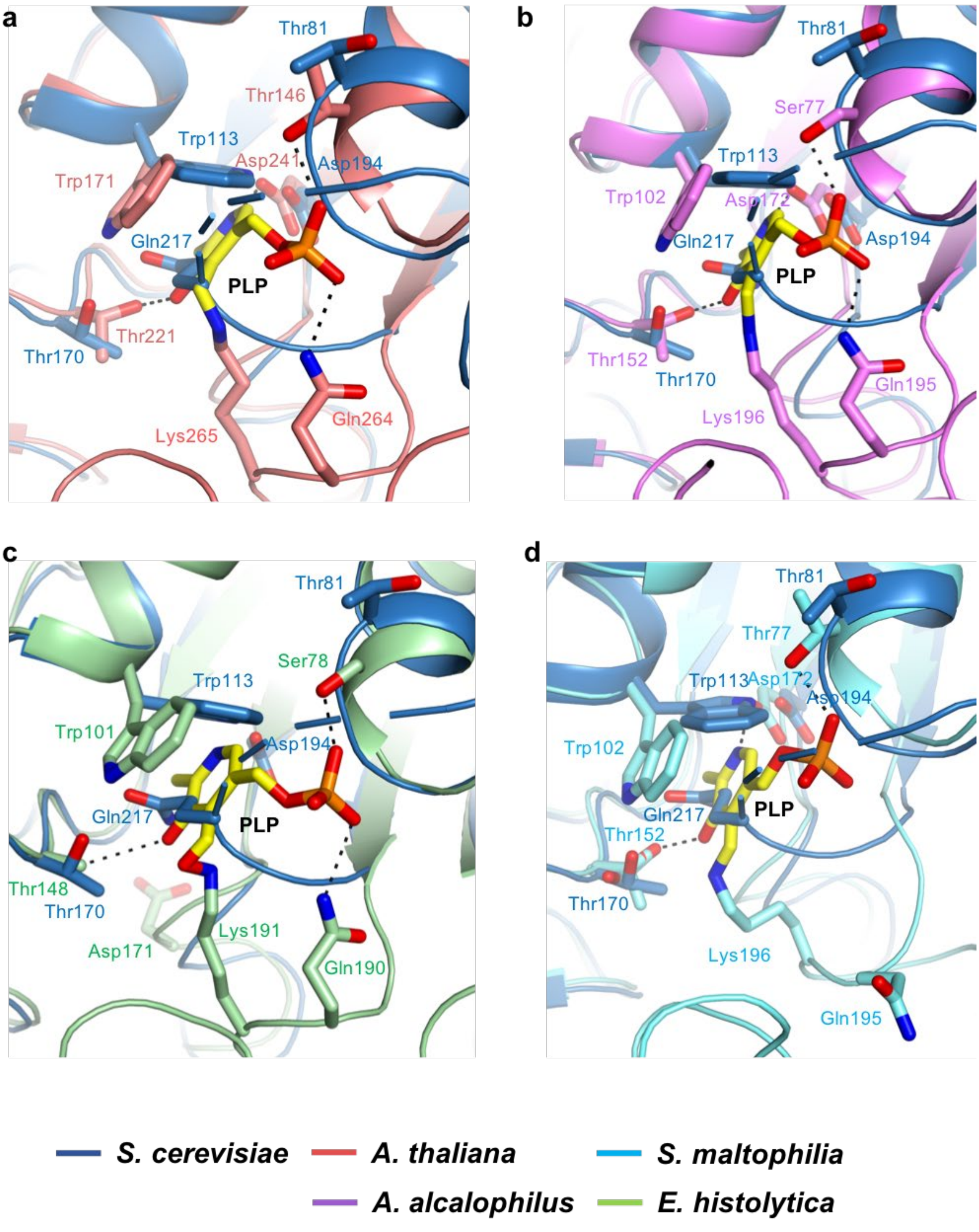

Figure S2. Comparison of the active site of *Sc*PSAT with its homologs: (**a**) Detailed view of the active site by superimposed structures of *Sc*PSAT and *At*PSAT-PLP complex. The *Sc*PSAT and *At*PSAT structures are colored blue and pink, respectively. Residues His44 and Arg45 are from a neighboring molecule of *Hs*PSAT. Covalently linked PLP with Lys265 bound in the *At*PSAT is colored in yellow; (**b**) Detailed view of the active site by superimposed structures of *Sc*PSAT and *Aa*PSAT-PLP complex. The *Sc*PSAT and *Aa*PSAT structures are colored blue and purple, respectively. Covalently linked PLP with Lys196 bound in the *Aa*PSAT is colored in yellow; (**c**) Detailed view of the active site by superimposed structures of *Sc*PSAT and *Eh*PSAT-PLP complex. The *Sc*PSAT and *Eh*PSAT structures are colored blue and green, respectively. Covalently linked PLP with Lys191 bound in the *Eh*PSAT is colored in yellow; (**d**) Detailed view of the active site by superimposed structures of *Sc*PSAT and *Sm*PSAT-PLP complex. The *Sc*PSAT and *Sm*PSAT structures are colored blue and cyan, respectively. Covalently linked PLP with Lys196 bound in the *Sm*PSAT is colored in yellow; Abbreviations: *Aa*, *Alkalihalobacillus alcalophilus*. *At*, *Arabidopsis thaliana*. *Eh*, *Entamoeba histolytica*. *Hs*, *Homo sapiens*. PLP, pyridoxal-5-phosphate. PSAT, 3-phosphoserine aminotransferase. *Sc*, *Saccharomyces cerevisiae*. *Sm*, *Stenotrophomonas maltophilia*.

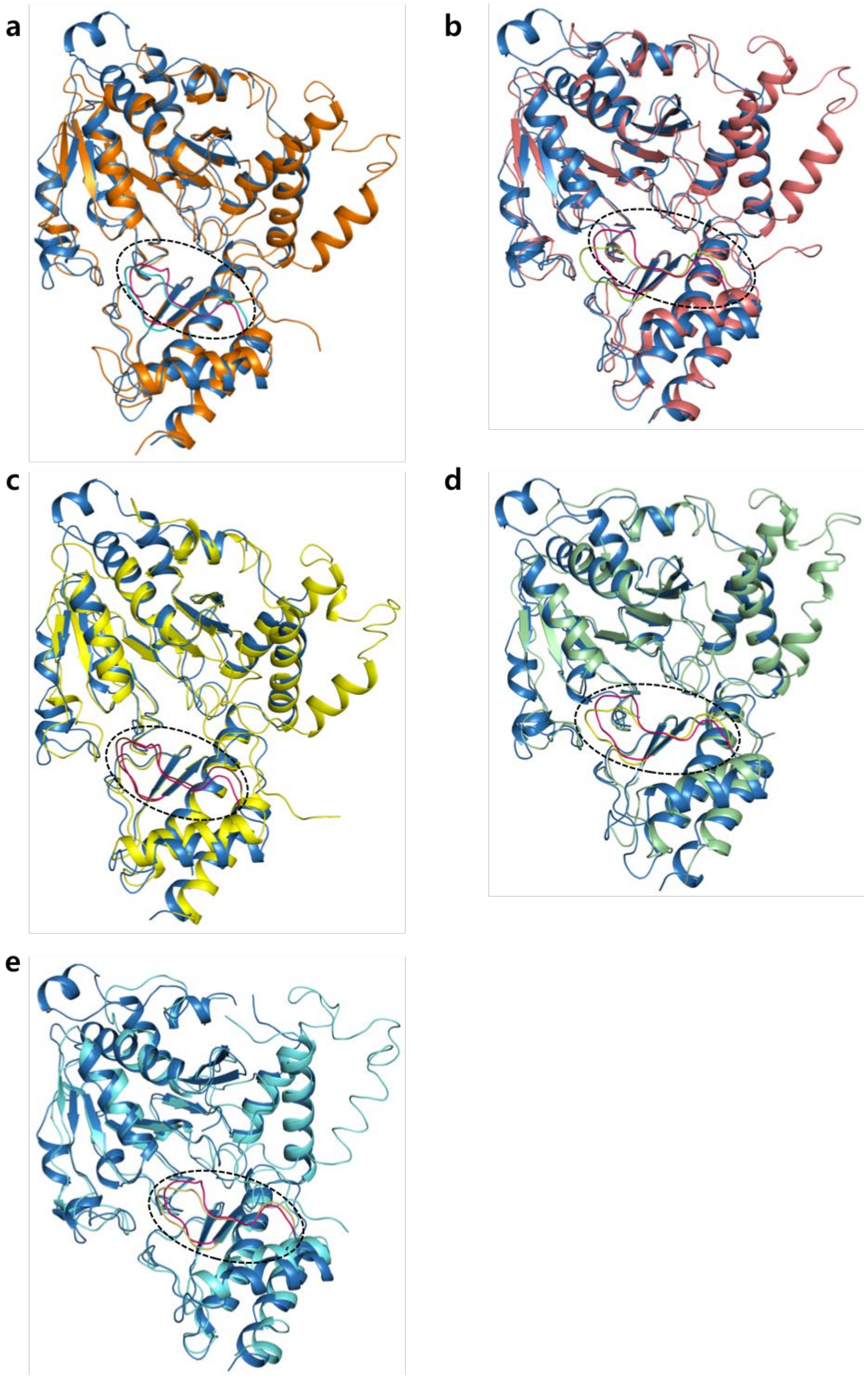

Figure S3. Structural comparison of the gate-keeping loop of *Sc*PSAT with its homologs: (**a**) Superposition of *Sc*PSAT (blue) and *Hs*PSAT (PDB ID: 3E77; orange) structures. The gate-keeping loops of *Sc*PSAT (red) and *Hs*PSAT (cyan) are highlighted by a black-dashed oval; (**b**) Superposition of the *Sc*PSAT (blue) and *At*PSAT (PDB ID: 6CZX; pink) structures. The gate-keeping loops of *Sc*PSAT (red) and *At*PSAT (green) are highlighted by a black-dashed oval; (**c**) Superposition of the *Sc*PSAT (blue) and *Aa*PSAT (PDB ID: 1W23; yellow) structures. The gate-keeping loops of *Sc*PSAT (red) and *Aa*PSAT (brown)are highlighted by a black-dashed oval; (**d**) Superposition of the *Sc*PSAT (blue) and *Eh*PSAT (PDB ID: 5YB0; light green) structures. The gate-keeping loops of *Sc*PSAT (red) and *Eh*PSAT (yellow) are highlighted by a black-dashed oval; (**e**) Superposition of the *Sc*PSAT (blue) and *Sm*PSAT (PDB ID: 6XDK; cyan) structures. The gate-keeping loops of *Sc*PSAT (red) and *Sm*PSAT (yellow) are highlighted by a black-dashed oval.

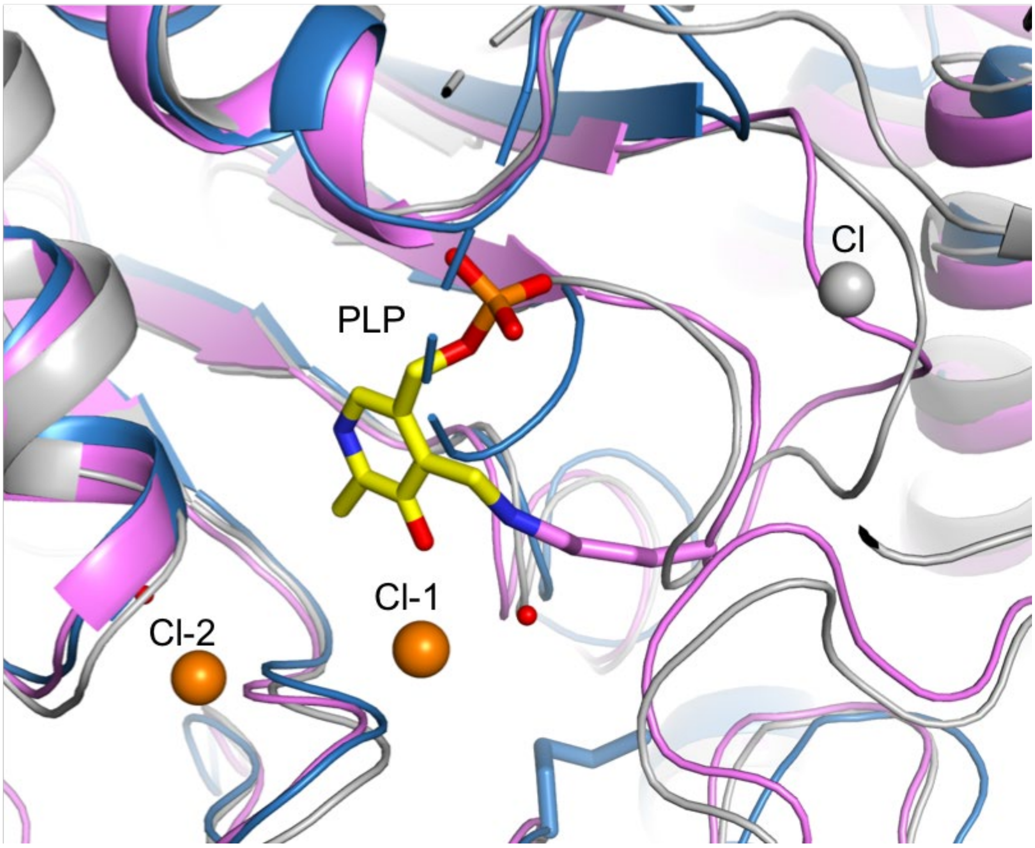

Figure S4. Distinct chloride ion binding in Stenotrophomonas maltophilia PSAT. The halide binding site of SmPSAT (grey) is different to ScPSAT (blue) and HsPSAT-PLP complex. The chloride ions of HsPSAT-PLP and SmPSAT are colored orange and grey, respectively. PLP-bound in HsPSAT is colored yellow.
